# New Diagnostic Model for the Differentiation of Diabetic Nephropathy From Non-Diabetic Nephropathy in Chinese Patients

**DOI:** 10.3389/fendo.2022.913021

**Published:** 2022-06-30

**Authors:** WeiGuang Zhang, XiaoMin Liu, ZheYi Dong, Qian Wang, ZhiYong Pei, YiZhi Chen, Ying Zheng, Yong Wang, Pu Chen, Zhe Feng, XueFeng Sun, Guangyan Cai, XiangMei Chen

**Affiliations:** ^1^ Department of Nephrology, The First Medical Center, Chinese PLA General Hospital, Chinese PLA Institute of Nephrology, State Key Laboratory of Kidney Diseases, National Clinical Research Center for Kidney Diseases, Beijing, China; ^2^ Beijing Computing Center, Beike Industry, Yongfeng Industrial Base, Beijing, China; ^3^ Department of Nephrology, Hainan Hospital of Chinese PLA General Hospital, the Hainan Academician Team Innovation Center, Sanya, China

**Keywords:** non-diabetic renal disease, diabetic nephropathies, diagnosis model, machine learning, renal biopsy

## Abstract

**Background:**

The disease pathology for diabetes mellitus patients with chronic kidney disease (CKD) may be diabetic nephropathy (DN), non-diabetic renal disease (NDRD), or DN combined with NDRD. Considering that the prognosis and treatment of DN and NDRD differ, their differential diagnosis is of significance. Renal pathological biopsy is the gold standard for diagnosing DN and NDRD. However, it is invasive and cannot be implemented in many patients due to contraindications. This article constructed a new noninvasive evaluation model for differentiating DN and NDRD.

**Methods:**

We retrospectively screened 1,030 patients with type 2 diabetes who has undergone kidney biopsy from January 2005 to March 2017 in a single center. Variables were ranked according to importance, and the machine learning methods (random forest, RF, and support vector machine, SVM) were then used to construct the model. The final model was validated with an external group (338 patients, April 2017–April 2019).

**Results:**

In total, 929 patients were assigned. Ten variables were selected for model development. The areas under the receiver operating characteristic curves (AUCROCs) for the RF and SVM methods were 0.953 and 0.947, respectively. Additionally, 329 patients were analyzed for external validation. The AUCROCs for the external validation of the RF and SVM methods were 0.920 and 0.911, respectively.

**Conclusion:**

We successfully constructed a predictive model for DN and NDRD using machine learning methods, which were better than our regression methods.

**Clinical Trial Registration:**

ClinicalTrial.gov, NCT03865914.

## 1 Introduction

As lifestyle changes, the number of diabetes mellitus (DM) patients increases globally, particularly in those with type 2 DM (T2DM) (1). The latest edition (10th edition) of the International Diabetes Federation Diabetes Atlas shows that 537 million adults are currently living with diabetes globally, and there are 140.9 million DM patients in China, which is the country with the largest number of DM patients (2). Diabetic nephropathy (DN) is one of the most important microvascular complications caused by DM, and approximately 30–40% of DM patients develop DN (3), while one-third of DN eventually develop into end-stage renal disease (ESRD) (4, 5). DM has become the major cause of ESRD worldwide (4). In 2018, ESRD was attributed to diabetes in Singapore (66.4%), Malaysia (66.2%), Qatar (63.9%), Hong Kong (52.0), and China (13.30%) (6, 7).

The disease pathology for DM patients with CKD may be DN, non-diabetic renal disease (NDRD), or DN combined with NDRD. Considering that the prognosis and treatment of DN and NDRD differ, their differential diagnosis is significant (8). Compared to DN, NDRD would have a better renal prognosis and longer survival in most cases. The 2007 Kidney Disease Outcomes Quality Initiative (KDOQI) guidelines are widely used for the clinical distinguish between DN and NDRD, but our previous study found that the specificity (40.63%) of the KDOQI guidelines was insufficient (9).

Some indicators, such as diabetic retinopathy (DR), DM course, and Hb could help in identifying DN and NDRD (10–12). However, the efficiency and accuracy of a single indicator may be insufficient. Currently, renal pathological biopsy is the gold standard for diagnosing DN and NDRD. However, its implementation in patients with diabetes-related CKD is subject to various contraindications, and the patient refused. Moreover, it is invasive and may cause complications.

In our previous study, we used the regression method to establish the distinguishing model (13, 14). Currently, machine learning methods have many advantages compared to traditional regression methods (15). In this study, we used support vector machine (SVM) and random forest (RF) methods to build new models (16, 17).

Using renal pathological biopsy as the gold standard, we established a noninvasive differential diagnostic model based on SMM and RF to distinguish DN and NDRD patients.

## 2 Materials and Methods

### 2.1 Patient Selection

This retrospective study included 1,030 consecutive patients with type 2 diabetes who required kidney biopsy at our institution from January 2005 to March 2017 for model development. Additionally, we screened 338 patients from April 2017 to April 2019 at our center for external validation. The variables used for external validation were the same as the variables in the development set. This study was attached to a registered clinical trial [ClinicalTrial.gov. Registry (NCT03865914)].

**Figure 1:**
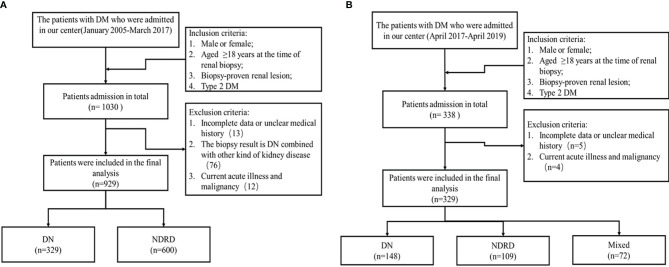
Patient screening process. **(A)** Screening process for the modeling group. **(B)** Screening process for the external validation group. Mixed: the biopsy result is DN combined with other kind of kidney disease.

The study protocol was approved by the Medicine Ethics Committee of the Chinese People’s Liberation Army General Hospital (Approval No. S2014-012-01). Each patient provided written informed consent before their participation in the study. All patients were screened according to the protocol shown in **Figure 1**. Patients were classified as having DN, NDRD, or DN combined with NDRD based on the results of the kidney biopsy. Patients with both DN and NDRD were excluded from the modeling population (13).

All patients underwent kidney biopsy after signing the informed consent form. The indications for kidney biopsy were consistent with our previous standards (14). The kidney biopsy indications for the suspected diagnosis of NDRD at our center were in accordance with those listed in the 2007 KDOQI guidelines (18).

Type 2 diabetes was defined according to the World Health Organization criteria (19). DN was diagnosed on the basis of histological characteristics, such as glomerular hypertrophy, thickened capillary basement membranes, diffuse mesangial expansion, nodular mesangial sclerosis, exudative lesions such as capsular drop or fibrin cap, mesangiolysis, capillary microaneurysm, or hyalinosis of afferent and efferent arterioles (20). NDRD was diagnosed on the basis of the classical criteria (21). The results were obtained independently from two pathologists. Discordant results were solved through a discussion.

### 2.2 Data Collection

Baseline characteristics and clinical parameters were collected from all patients before kidney biopsy. Baseline characteristics included age, sex, body mass index (BMI), blood pressure (BP), and medical history of DM and/or hypertension. Laboratory data obtained included measurements of HbA1c, hemoglobin (Hb), serum creatinine (sCr), serum albumin (ALB), estimated glomerular filtration rate (eGFR), and serum lipid levels. Variable definitions have been provided in previous studies (9).

### 2.3 Variable Selection and Treatment of Missing Data

The variable importance of all 49 candidate variables (listed in **Supplementary Table 1**) was ranked using the RF classification method. Missing values were imputed by predictive mean matching. The Gini index was used for decisions related to variable ranking or node splitting. The importance and clinical significance of all variables were evaluated to rank the variables.

### 2.4 Model Building

**Figure 2:**
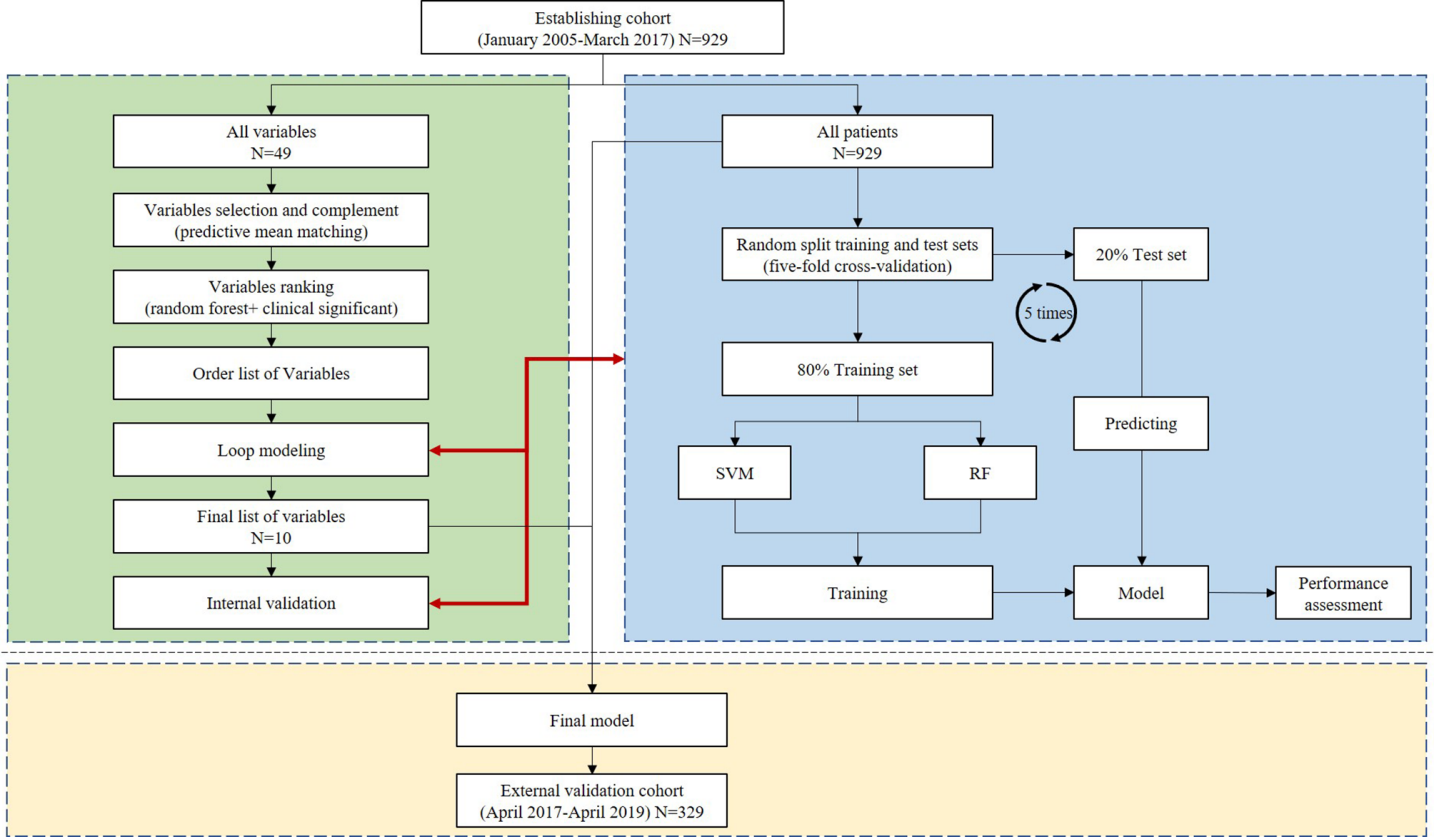
Analysis flow for the development and evaluation of the models.

All processes are shown in **Figure 2**. For new model development, SVM and RF methods were used.

Model construction steps: the entire modeling used RF and SVM models. RF modeling used the ranger module in the R language (parameter: importance = “impurity”), and SVM modeling used the kernellab module in the R language (parameters: kernel = “rbfdot”; C = 2, cross = 5). The detailed modeling steps are described as follows:

(1) Random forest and support vector machine modeling were performed for 929 samples in the training set (including 49 variables in total) (note: the steps were performed on the RF and SVM models respectively).
(2) The RF model was used to screen 49 variables, and finally, 10 features were determined as variables in the final modeling.
(3) The 929 samples of the training set and the 10 final selected variables were used to perform five-fold cross modeling (80% of the randomly divided data is used as the training set, and 20% as the verification set), and the modeling results were evaluated.
(4) Modeling was performed with all 929 samples.
(5) The final model was constructed using all the samples, and the 329 externally reserved samples were used as the test data set; model evaluation was performed, and the receiver operating characteristic (ROC) curve was drawn.

#### 2.4.1 Random Forest

The RF machine learning algorithm is an ensemble tree method that can generate many regression trees to detect interactions. At each split, a candidate variable that maximizes the difference in cumulative hazard between the daughter nodes was chosen, and the splitting stopped at the terminal nodes when the data at hand can be split such that each terminal node has at least one unique outcome (22).

#### 2.4.2 Support Vector Machine

The SVM algorithm was originally proposed by Vapnik and colleagues in 1963 (23). It creates a decision boundary, known as the hyperplane, between two classes (24). The input variables entered were either continuous or categorical data in the SVM, whereas the output variables entered were binary data. The dataset was further divided into training and validation subsets. The variables were entered into the classifier to yield the final model.

#### 2.4.3 Logistic Regression

Logistic regression was performed using SPSS version 20.0 (IBM Corp., Armonk, NY). Accuracy and effectiveness were calculated using the formulas previously established by Zhou Jianhui (2008) and Liu Moyan (2014) at our center (9, 10), which were abbreviated as model-2008 and model-2014. The two formulas are listed in **Supplementary Table 2**.

#### 2.4.4 Other Statistical Analyses

The remaining statistical analyses were performed using SPSS version 20.0 (IBM Corp., Armonk, NY) and R version 3.5.1 (R Foundation for Statistical Computing, Vienna, Austria) (25). Clinical and laboratory features were compared between the groups through analysis of variance. This information is presented as means and standard deviations. Kruskal–Wallis tests were performed to compare medians and interquartile ranges. Pearson’s chi-square test was used for numbers and proportions; the test was used to compare performances among models. A two-tailed P-value of <0.05 was considered statistically significant.

## 3 Results

### 3.1 Trial Population

In total, 929 patients were included in the development set and assigned to one of the two groups (DN and NDRD), based on the outcomes of the kidney biopsy. Patient characteristics are shown in **Supplementary Table 3**. The mean age (at the time of kidney biopsy) was 51.34 ± 10.02 (range, 19–85) years. Patients in the DN group had higher BP than those in the NDRD group (p <0.05). Patients with DN were more likely to experience DR and cardiovascular and cerebrovascular diseases (CCVD) than those with NDRD. Compared with patients with DN, patients with NDRD were more likely to have higher levels of Hb, BMI, ALB level, eGFR, Ucr, serum lipid, total cholesterol, and lower levels of SBP, DBP, MAP, pulse pressure, FBG, 24-hour proteinuria, and BUA.

### 3.2 Pathological Results of the NDRD Group

The results of the kidney biopsy indicated that 329 (35.41%) and 600 (64.59%) patients had DN and NDRD, respectively. Numerous pathological subtypes were observed in the NDRD group; membranous nephropathy was frequently observed (32.33%), followed by IgA nephropathy (30.83%), and then mesangial proliferative glomerulonephritis (6.00%) (**Supplementary Table 4**).

### 3.3 Importance Ranking

Various predictors were ranked based on their order of importance, using the RF method. DR was found to be the most important, followed by the duration of DM and then Hb, pulse pressure (PP), sCr, and ALB levels (**Supplementary Table 5** lists the top 21).

### 3.4 Traversal Modeling and Selected Markers

We exhausted all combinations of variables (variables ranged from 6 to 12) for RF and SVM, and the best combinations are listed in **Supplementary Table 6**. Moreover, we observed that the increase in performance with ≥10 markers was marginal (**Supplementary Figure 1**). We then obtained the best combination of the variables, which consisted of the following: DR, DM course, Hb, PP, sCr, ALB, total cholesterol (TC) levels, sudden onset of heavy proteinuria, hematuria, and family history of DM.

### 3.5 Model Building

The performances of RF and SVM are shown in **Tables 1**, **2**. The AUCROC for RF reached 0.953 (0.939–0.967), and that for the SVM method reached 0.947 (0.931–0.963).

**Table 1:** Five-fold cross validation for the random forest method with 10 variables.

RF	Accuracy	Sensitivity	Specificity	PPV	NPV	Balanced accuracy	AUCROC
1	0.908	0.894	0.916	0.855	0.940	0.905	0.946
2	0.881	0.746	0.951	0.887	0.879	0.848	0.946
3	0.903	0.889	0.91	0.836	0.941	0.899	0.974
4	0.849	0.848	0.849	0.725	0.922	0.848	0.938
5	0.860	0.844	0.868	0.771	0.913	0.856	0.960
Average	0.880	0.844	0.899	0.815	0.919	0.871	0.953
SD	0.026	0.059	0.040	0.066	0.025	0.028	0.014

AUCROC, area under the receiver operating characteristic curve; NPV, negative predictive value; PPV, positive predictive value; RF, random forest.

**Table 2:** Five-fold cross-validation for the support vector machine method with 10 variables.

SVM	Accuracy	Sensitivity	Specificity	PPV	NPV	Balanced accuracy	ROC AUC
1	0.892	0.877	0.900	0.826	0.931	0.889	0.948
2	0.870	0.723	0.950	0.887	0.864	0.837	0.928
3	0.908	0.868	0.932	0.881	0.924	0.900	0.972
4	0.865	0.867	0.864	0.754	0.931	0.865	0.940
5	0.881	0.875	0.884	0.8	0.93	0.880	0.947
Average	0.883	0.842	0.906	0.829	0.916	0.874	0.947
SD	0.017	0.067	0.035	0.056	0.029	0.024	0.016

AUC ROC, area under the receiver operating characteristic curve; NPV, negative predictive value; PPV, positive predictive value; SVM, support vector machine.

### 3.6 External Validation

In total, 329 patients were enrolled for external validation, and their characteristics are presented in **Supplementary Table 7**. Validations were performed using the same variables as in the development set. We validated the four models under two conditions. First, the calculations were performed under ideal conditions (isolated DN vs. isolated NDRD). Second, the calculations were performed under actual conditions (DN patients vs. non-DN patients [NDRD with and without DN]) (9, 26). Regarding DN vs. NDRD, the AUCROC for the RF reached 0.920, and the SVM reached 0.911; model-2008 reached 0.886, and model-2014 reached 0.917. For DN vs. non-DN patients, the AUCROC for RF also reached 0.855, and SVM reached 0.846; model-2008 reached 0.821, and model-2014 reached 0.841 (**Table 3**; **Supplementary Figure 2**).

**Table 3:** Performance for SVM and other models in external validation.

	Models	Sensitivity	Specificity	PPV	NPV	AUCROC
Isolated DN vs. isolated NDRD	SVM	0.867	0.889	0.926	0.807	0.911
	RF	0.905	0.864	0.899	0.872	0.920
	Model-2008	0.893	0.706	0.730	0.881	0.886
	Model-2014	0.858	0.853	0.899	0.798	0.917
Isolated DN vs. non-DN	SVM	0.717	0.890	0.892	0.713	0.846
	RF	0.735	0.899	0.899	0.735	0.855
	Model-2008	0.732	0.765	0.703	0.790	0.821
	Model-2014	0.688	0.883	0.892	0.669	0.841

AUCROC, area under the receiver operating characteristic curve; NPV, negative predictive value; PPV, positive predictive value; SVM, support vector machine; RF, random forest; DN, diabetic nephropathy; NDRD, non-diabetic nephropathy.

The comparison among the four models showed that RF and SVM do have better diagnostic performance than logistic regression models (**Supplementary Table 8**). The chi-square test also showed that there was no significant difference for each model between the two conditions (isolated DN vs. isolated NDRD; DN vs. non-DN), which means that these models also have good performances in the real world (**Supplementary Table 9**).

## 4 Discussion

Although some CKD patients have a clear history of diabetes and other complications (such as DR), which are clinically suspected to be diabetic nephropathy, there are still some kidney biopsy results of NDRD or DN combined with NDRD. The incidence of DN and NDRD varies greatly in different studies. The prevalence of DN ranges from 6.5 to 94% with an average of 41.3% (27–29); NDRD ranges from 3 to 93.5% with an average of 40.6% (29–31); and DN with NDKD ranges from 0 to 45.5%, with an average of 18.1% (29) in the CKD patients with T2DM history. Our study showed that 64.58% of biopsied type 2 diabetic patients were diagnosed with NDRD.

In our study, membranous nephropathy (MN) was the most common type, accounting for 32.33% of all NDRD cases, followed by IgA nephropathy, which accounted for 30.83%. In previous studies, MN (7 to 35%) was the most common cause of NDRD (32, 33), ranging from 7 to 35% (34–37). Some studies have also shown IgA nephropathy was the most common type of NDRD, which ranged from 3 to 59% (31, 38–40). Some researchers found that the prevalence of membranous nephropathy increased from 12 to 24% during the last decades (41), which might be due to the impact of air pollution and other factors (42).

DR, DM course, Hb levels, PP, and hematuria were among the most important indicators of diagnosis through importance ranking in our study. Same with the previous studies, which reported that the absence of DR (43), hematuria (44), and shorter course of DM (45) are risk factors for NDRD, while lower hemoglobin (32) and high BP (44) levels are risk factors for DN. We recently listed the published models for differential diagnosis of DN and NDRD (**Supplementary Table 10**) (13, 14, 46–48), which are most based on logistic regression analyses. Compared with the previous studies, we made use of larger samples and new methods to build our modes, and we compared two of them with our models in external groups.

DR ranked first in all indicators; DR was a co-occurrence in 79.3% of DN patients in this study. Previous studies ranged from 37 to 84% (49–51). In most cases, the kidney and eye were damaged at the same time for DM patients. It used to be emphasized that the absence of DR indicates a high likelihood of NDRD but not always, for organ heterogeneity, and they might have different protective factors. Some patients did not always have DR and DN at the same time. Studies showed that 10–70% of biopsied DN patients did not have DR, and 6–57.4% of biopsied NDRD patients had DR (48, 52–55). Thus, the single DR index may make mistakes in verifying DN and NDRD.

The presence of microhematuria in diabetic patients with proteinuria may range from 11 to 76–78% (11, 56, 57), while that in DN patients ranges from 5 to 75%. In our study, the prevalence of hematuria in the NDRD group was significantly higher than that in the DN group (5.8% versus 24.2%). It was believed that hematuria was of great significance in distinguishing between DN and NDRD. But recently, a meta-analysis concluded that type 2 diabetes patients presenting with hematuria may be slightly more likely to develop NDRD (58).

In our study, the DN group tended to have more severe hypertension than the NDRD group. The systolic and diastolic blood pressures (SBP and DBP) of the DN group were higher than those of the NDRD group, and this result is consistent with that in our previous study, which reported that lower BP was a good predictor of NDRD (10, 13, 59). In our study, lower PP was a better predictor of NDRD than both SBP and DBP in our study. DN, as the microvascular disease of DM, has many typical vascular lesions, such as arteriolar hyalinosis, arteriosclerosis, and the presence of large vessels (60), which might lead to a higher PP in DN patients.

Kidney biopsy is the gold standard method for diagnosis, but it cannot be performed on all patients because of contraindications (anticoagulation, active bleeding, and unilateral nephrectomy) and reluctance to undergo biopsy. As it is an invasive technique with certain risks, such as hematuria and perirenal hematoma, arterial embolization might be required (61, 62). Moreover, there is no consensus on the indications for biopsy in DM patients (63, 64). It is believed that the main purpose of biopsy is to detect cases of NDRD (63, 64). Nephrotomy was implemented to clarify its pathological type and implement earlier intervention (62). The rates of renal biopsies performed in patients with DN are variable, ranging from 12 to 80% (65–68). Thus, the limited accuracy of a single indicator and the implementation of kidney biopsy make it of great significance to establish a non-invasive differential diagnosis model for the diagnosis of DN and NDRD.

The previous non-invasive identification guidelines widely used internationally are the 2007 KDOQI guidelines (18), which were evaluated in Chinese patients and found that many of the predictors were binary categorical variables. The low specificity of the guidelines renders them not suitable for diagnostic criteria (9). So we built the models using the logistic regression method in 2008 and 2014 (13, 14). Recently, Jiang et al. [(47), n = 302)], Yang et al. [(46), n = 213)], and García-Martín [(48), n = 207)] also used the logistic regression method to build the diagnostic models, and all the models found the index like DR and DM course are important. In this study, we developed new models with machine learning and compared them with our previous models established in 2008 and 2014. The results showed that RF and SVM were superior to the traditional method.

Machine learning has been used in many diabetes-related CKD studies, such as genotype–phenotype risk patterns (69) and ensemble feature selection for clinical markers (70). It is also used to select the risk factors for diabetes-related CKD development (70, 71) or to construct a model to detect the progression of diabetes-related CKD (72). In this study, we used it to distinguish between DN and NDRD.

This study had several limitations. First, the time span of patients included is quite large (2005–2017); thus, the disease patterns for DM patients combined with CKD may change over this long period. Therefore, we built a new model using a new population (2015–2017), and it was verified externally. The 2015–2017 model did not show better performance (**Supplementary Table 10**) than the previous model (2005–2017), which indicates that the large time span of enrollment has little influence. Second, the model mainly included clinical predictors rather than some new predictors, which may limit the further improvement of the model accuracy. Finally, this is a retrospective and single-center study in a developing country like China. It may not be suitable for other countries.

Despite the above limitations, this model has provided a new method for the clinical differentiation between DN and NDRD. Our research has many advantages: 1. a large sample size avoids bias considerably; 2. all parameters were routine clinical variables that can be easily obtained and used by clinicians; 3. external verification shows that the models have low learning and test errors; 4. to enhance the effect of its practical application, we also verified patients with DN combined with NDRD, which indicated that the model has good feasibility in the real world; and 5. kidney biopsy results are the gold standard for verifying the accuracy of the models.

In conclusion, the main purpose of this study was to establish a noninvasive method to differentially diagnose DN and NDRD. As a result, fewer kidney biopsies can be performed, reducing the suffering and costs for patients.

## Data Availability Statement

The original contributions presented in the study are included in the article/**Supplementary Material**. Further inquiries can be directed to the corresponding author.

## Ethics Statement

Written informed consent was obtained from the individual(s) for the publication of any potentially identifiable images or data included in this article.

## Author Contributions

WZ: Design of the experiment, data statistics, and model building. XL: Data collection, statistical analysis, and model building. ZD and QW: Data collection. ZP: Statistical analysis and model building. YC, YW, and YZ: Collecting clinical data. PC and ZF: Kidney biopsy. XS, GC, and XC: Design of the experiment. All authors listed have made a substantial, direct, and intellectual contribution to the work and approved it for publication.

## Funding

This study was supported by the National Natural Science Foundation Of China (32141005), the Science & Technology Project of Beijing China (no. D171100002817002), the Beijing Municipal Science and Technology project (no. Z181100001918015), the National Basic Research Program of China (no. 2015CB553605), the National Key R&D Program of China (Nos. 2016YFC1305500, 2016YFC1305503), the Natural Science Foundation of China (Nos. 81601211, 8160050078), and the Specialized Scientific Program of the Hainan Province Academician Innovation Platform (YSPTZX202026).

## Conflict of Interest

The authors declare that the research was conducted in the absence of any commercial or financial relationships that could be construed as a potential conflict of interest.

## Publisher’s Note

All claims expressed in this article are solely those of the authors and do not necessarily represent those of their affiliated organizations, or those of the publisher, the editors and the reviewers. Any product that may be evaluated in this article, or claim that may be made by its manufacturer, is not guaranteed or endorsed by the publisher.

## Abbreviations

AUCROC, Area under the receiver operating characteristic curve; BP, Blood pressure; BMI, Body mass index; CKD, Chronic kidney disease; DM, Diabetes mellitus; DN, Diabetic nephropathy; DR, Diabetic retinopathy; Hb, Hemoglobin; KDOQI, Kidney Disease Outcomes Quality Initiative; NDRD, Nondiabetic renal disease; RF, Random forest; ALB, Serum albumin; sCr, Serum creatinine; SVM, Support vector machine.
