# Supplementary figures and images for: New Diagnostic Model for the Differentiation of Diabetic Nephropathy From Non-Diabetic Nephropathy in Chinese Patients
Source: Front Endocrinol (Lausanne). 2022 Jun 30;13:913021. doi: 10.3389/fendo.2022.913021 (PMC9279696; doi:10.3389/fendo.2022.913021)

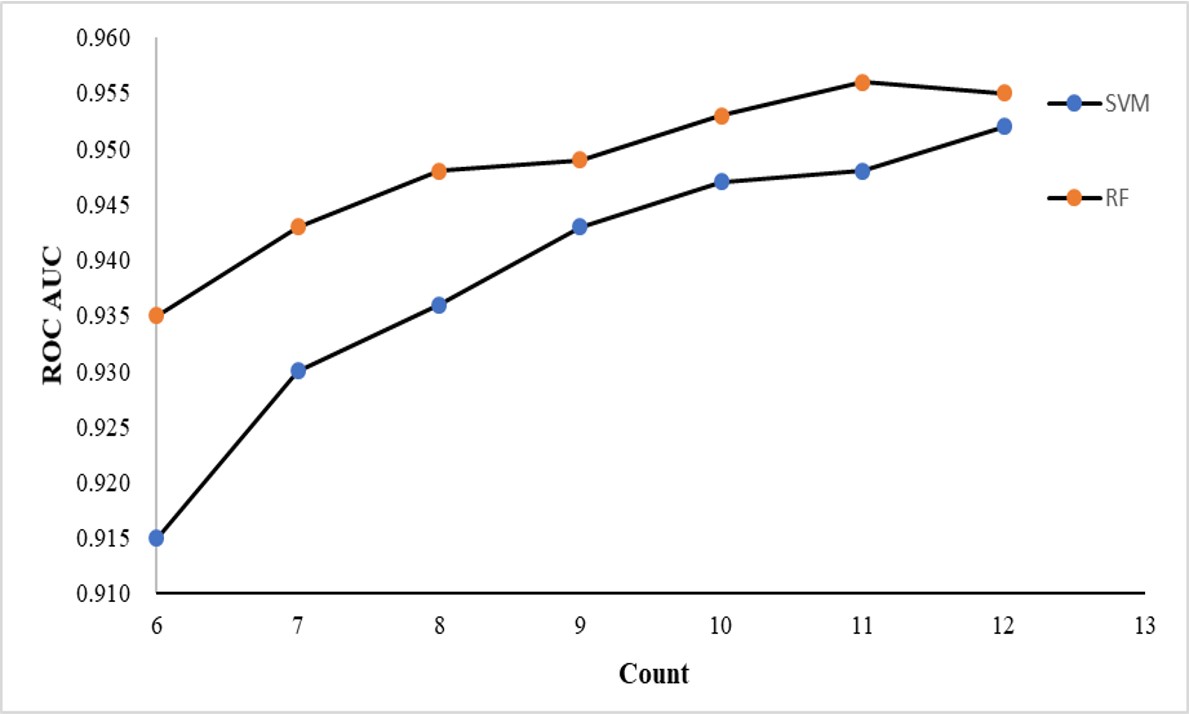

Supplement: Supplementary file 1 [file Image_1.jpeg]

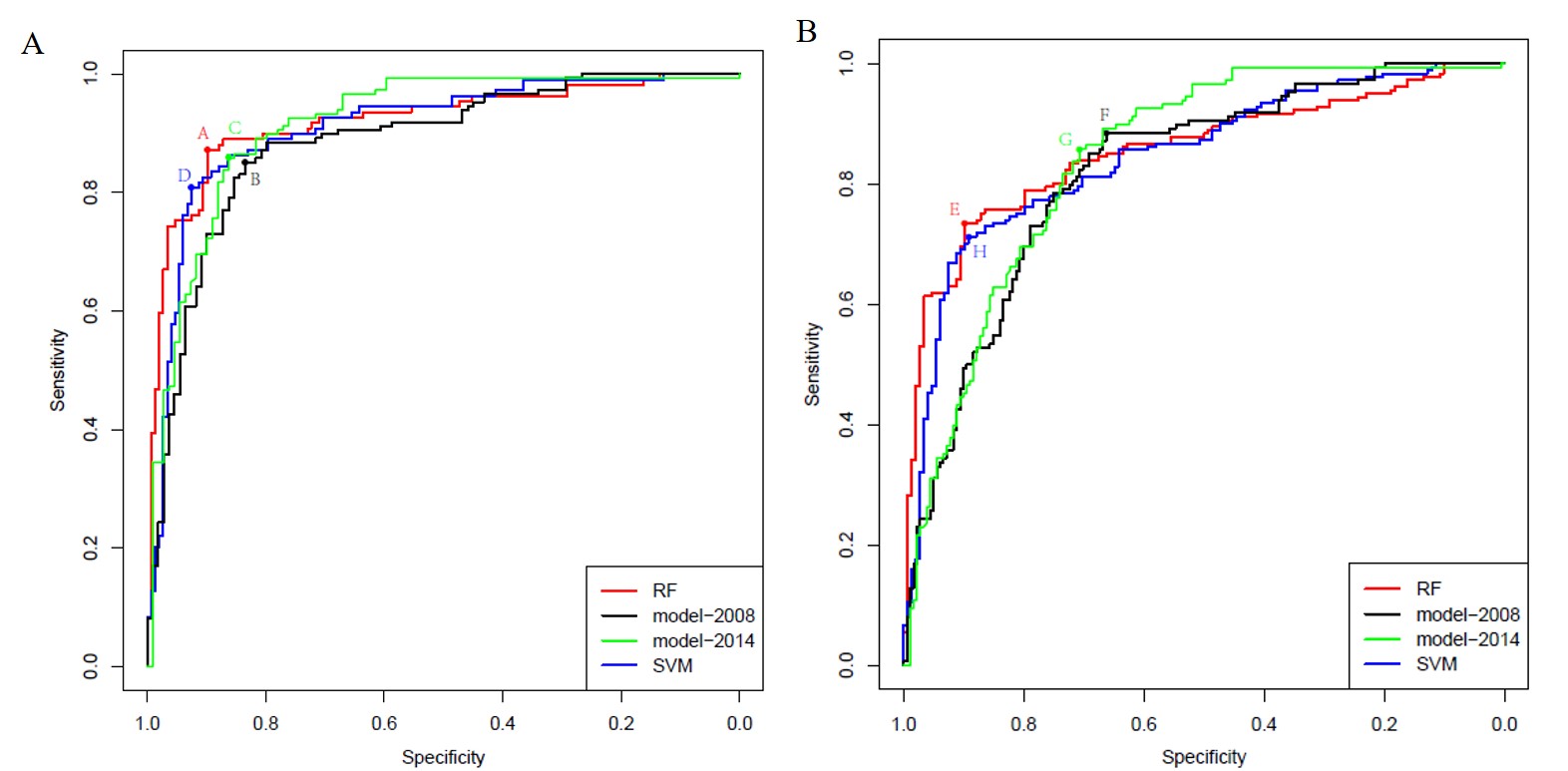

Supplement: Supplementary file 2 [file Image_2.jpeg]
