# Supplementary material for: New Diagnostic Model for the Differentiation of Diabetic Nephropathy From Non-Diabetic Nephropathy in Chinese Patients
Source: Front Endocrinol (Lausanne). 2022 Jun 30;13:913021. doi: 10.3389/fendo.2022.913021 (PMC9279696; doi:10.3389/fendo.2022.913021)
Supplement: Supplementary file 3 [file Table_1.docx]

**New diagnostic model for the differentiation of diabetic nephropathy from non-diabetic nephropathy in Chinese patients**

**Supplementary files**

## Tables

| Supplementary Table 1. All screened variables | | |
| --- | --- | --- |
| Serial number | Variables | Variable type |
| 1 | Age (years) | Continuous variable |
| 2 | SBP (mmHg) | Continuous variable |
| 3 | DBP (mmHg) | Continuous variable |
| 4 | MAP (mmHg) | Continuous variable |
| 5 | Pulse pressure (mmHg) | Continuous variable |
| 6 | Course of diabetes (m) | Continuous variable |
| 7 | Course of kidney disease (m) | Continuous variable |
| 8 | Course of proteinuria (m) | Continuous variable |
| 9 | Course of hypertension (m) | Continuous variable |
| 10 | HbA1C (%) | Continuous variable |
| 11 | BMI (kg/m^2^) | Continuous variable |
| 12 | Triglyceride (mmol/L) | Continuous variable |
| 13 | uCr (mmol/L) | Continuous variable |
| 14 | ALB (g/L) | Continuous variable |
| 15 | 24-hr proteinuria (g/24 h) | Continuous variable |
| 16 | eGFR (mL/min1#73/ m^2^) | Continuous variable |
| 17 | UOSM (mOsm/L) | Continuous variable |
| 18 | BUA (µmol/L) | Continuous variable |
| 19 | Hb (g/L) | Continuous variable |
| 20 | TC (mmol/L) | Continuous variable |
| 21 | Scr (µmol/L) | Continuous variable |
| 22 | FBG (mmol/L) | Continuous variable |
| 23 | Hematuria (n/HP) | Continuous variable |
| 24 | DR grading | Ordinal categorical variables |
| 25 | AKI grading | Ordinal categorical variables |
| 26 | Albuminuria (1=macro, 2=micro, 0=0) | Ordinal categorical variables |
| 27 | HBP grading | Ordinal categorical variables |
| 28 | HBP | Binary variable |
| 29 | NS | Binary variable |
| 30 | CKD | Binary variable |
| 31 | DR | Binary variable |
| 32 | CCVD | Binary variable |
| 33 | Smoke | Binary variable |
| 34 | Drink alcoholic beverages | Binary variable |
| 35 | Family history of DM | Binary variable |
| 36 | Family history of HBP | Binary variable |
| 37 | Family history of kidney disease | Binary variable |
| 38 | Sudden onset of heavy proteinuria | Binary variable |
| 39 | Hematuria | Binary variable |
| 40 | Systemic disease | Binary variable |
| 41 | DM <5 years | Binary variable |
| 42 | GFR >60 mL/min/1.73 m^2^, 24-h proteinuria >3.5 g/24h | Binary variable |
| 43 | AKI | Binary variable |
| 44 | Sex (male=1) | Binary variable |
| 45 | RH | Binary variable |
| 46 | Hyperlipidemia | Binary variable |
| 47 | High-cholesterol | Binary variable |
| 48 | Hypertriglyceride | Binary variable |
| 49 | Hyperuricemia | Binary variable |

AKI, acute kidney injury; ALB, albumin; BMI, body mass index; BUA, blood uric acid; Hb, hemoglobin; CCVDs, cardiovascular and cerebrovascular diseases; CKD, chronic kidney disease; DBP, diastolic blood pressure; DR, diabetic retinopathy; eGFR, estimated glomerular filtration rate; FBG, fasting blood glucose; HbA1c, hemoglobin A1C; MAP, mean arterial pressure; NS, nephrotic syndrome; RH, refractory hypertension; SBP, systolic blood pressure; sCr, serum creatine; TC, total cholesterol; uCr, urine creatine; UOSM, urine osmotic pressure

| Supplementary Table 2 Two models developed previously in our center | | |
| --- | --- | --- |
|  | Model-2008[1] | Model-2014(Liu Moyan)[2] |
| Models | *P_DN_* = exp (−13*.*5922 + 0*.*0371Dm+0*.*0395Bp+0*.*3224Gh − 4*.*4552Hu + 2*.*9613Dr)*/*[1 + exp(−13*.*5922 + 0*.*0371Dm + 0*.*0395Bp+0*.*3224Gh − 4*.*4552Hu + 2*.*9613Dr)] | *P*_DN_ = exp (0.846 + 0.022Dm + 0.033 Bp + 2.050 Gh–2.664 Hu–0.078 Hb + 2.942Dr)/[1 + exp (0.846 + 0.022 Dm + 0.033 Bp + 2.050Gh–2.664 Hu–0.078 Hb + 2.942 Dr)] |
| Variables | Dm, course of diabetes; Bp, SBP; Gh, HbA1C; Hu, hematuria; Hb, hemoglobin; Dr, diabetic retinopathy | Dm, course of diabetes; Bp, systolic blood pressure; Gh, HbA1C (1 HbA1c ≥ 7%, 0 < 7%); Hu, hematuria (1 urine RBC>10/HP, 0 < 10/HP); Hb, Hemoglobin; Dr, diabetic retinopathy |

| Supplementary Table 3. Clinical parameters of patients enrolled in the study | | | |
| --- | --- | --- | --- |
| Variables | DN (n=329) | NDRD (n=600) | *P*-value |
| Sex (male = 1) | 228(69.30%) | 380(63.30%) | 0.076 |
| Age (year) | 51.34±10.02 | 50.24±11.54 | 0.130 |
| SBP (mmHg) | 182.17±22.95 | 161.38±26.44 | <0.001 |
| DBP (mmHg) | 101.87±13.78 | 98.65±18.07 | <0.001 |
| MAP (mmHg) | 128.65±14.65 | 119.58±19.43 | <0.001 |
| Pulse pressure (mmHg) | 80.27±19.90 | 62.69±18.19 | <0.001 |
| HBP (%) | 319(97.0%) | 502(83.7%) | <0.001 |
| NS (%) | 109(33.1%) | 232(38.7%) | 0.094 |
| DR (%) | 261(79.3%) | 71(11.8%) | <0.001 |
| CCVD (%) | 101(30.7%) | 101(16.8%) | <0.001 |
| Smoke (%) | 145(44.1%) | 214(35.7%) | 0.012 |
| Drink alcoholic beverages (%) | 139(42.2%) | 202(33.7%) | 0.009 |
| Course of diabetes (m) | 144(72,192) | 11(3,24) | <0.001 |
| Course of hypertension (m) | 24(4.5,60) | 16(0,84) | 0.030 |
| HbA1c (mmol/mol) | 52.98±15.98 | 51.25±14.23 | 0.091 |
| HbA1c (%) | 7.00±1.46 | 6.84±1.30 | 0.083 |
| FBG (mmol/L) | 6.74±2.42 | 6.09±1.88 | <0.001 |
| Hb (g/L) | 112.02±21.04 | 133.33±21.87 | <0.001 |
| BMI (kg/m^2^) | 26.29±3.63 | 27.26±3.86 | <0.001 |
| Ucr (mmol/L) | 5.53±2.54 | 6.48±4.79 | <0.001 |
| ALB (g/L) | 32.48±5.84 | 33.27±9.21 | 0.004 |
| 24-hour proteinuria (g/24 h) | 3.84(2.13,6.15) | 2.50(1.00,4.94) | <0.001 |
| eGFR (mL/min1#73/m^2^) | 55.60±28.34 | 77.43±32.31 | <0.001 |
| BUA (µmol/L) | 368.36±82.52 | 360.22±110.86 | 0.028 |
| TC (mmol/L) | 5.18±1.61 | 5.79±2.31 | 0.002 |
| Triglyceride (mmol/L) | 2.13±1.63 | 2.59±1.95 | <0.001 |
| Scr (µmol/L) | 124.10(92.40,183.60) | 86.45(65.20,118.50) | <0.001 |
| Family history of DM (%) | 145(44.1%) | 156(26.0%) | <0.001 |
| Family history of HBP (%) | 82(24.9%) | 181(30.2%) | 0.09 |
| Sudden onset of heavy proteinuria (%) | 44(13.4%) | 245(40.8%) | <0.001 |
| Hematuria (%) | 19(5.8%) | 145(24.2%) | <0.001 |
| Systemic disease (%) | 31(9.4%) | 92(15.3%) | 0.011 |

SBP, systolic blood pressure; DBP, diastolic blood pressure; MAP, mean arterial pressure; NS, nephrotic syndrome; DR, diabetic retinopathy; CCVDs, cardiovascular and cerebrovascular diseases; HbA1c, hemoglobin A1C; FBG, fasting blood glucose; BMI, body mass index; ALB, albumin; eGFR, estimated glomerular filtration rate; BUA, blood uric acid; TC, total cholesterol; DM, diabetes mellitus; DN, diabetic nephropathy; NDRD, non-diabetic nephropathy; Scr, serum creatinine

| **Supplementary Table 4. Pathological results of the non-diabetic nephropathy group** | | |
| --- | --- | --- |
| Pathological diagnosis | Number | % |
| Membranous nephropathy | 194 | 32.33 |
| IgA nephropathy | 185 | 30.83 |
| Mesangial proliferative glomerulonephritis | 36 | 6.00 |
| Focal segmental glomerular sclerosis | 35 | 5.83 |
| Obesity-related glomerulopathy | 31 | 5.17 |
| Hypertensive renal damage | 28 | 4.67 |
| Hepatitis B virus-associated glomerulonephritis | 18 | 3.00 |
| Anaphylactic purpura nephritis | 18 | 3.00 |
| Minimal change glomerulopathy | 16 | 2.67 |

| **Supplementary Table 5. Various indicators ranked in order of importance** | |
| --- | --- |
| Importance ranking | Indicator |
| 1 | DR |
| 2 | Course of DM |
| 3 | Hb |
| 4 | PP |
| 5 | Course of DM <5 years |
| 6 | sCr |
| 7 | ALB |
| 8 | SBP |
| 9 | eGFR |
| 10 | TC |
| 11 | MAP |
| 12 | FBG |
| 14 | 24-h proteinuria |
| 15 | Course of hypertension |
| 16 | Sudden onset of heavy proteinuria |
| 17 | Age |
| 18 | BUA |
| 19 | Hematuria |
| 20 | Family history of DM |
| 21 | Systemic disease |

ALB, albumin; BUA, blood uric acid; DM, diabetic mellitus; DR, diabetic retinopathy; eGFR, estimated glomerular filtration rate; FBG, fasting blood glucose; Hb, hemoglobin; MAP, mean arterial pressure; PP, pulse pressure; SBP, systolic blood pressure; sCr, serum creatine; TC, total cholesterol

| **Supplementary Table 6. Best combinations of variables at each number of indices (from 6 to 12) for the random forest methods and support vector machine** | | | |
| --- | --- | --- | --- |
| Number of variables | Random forest | Support vector machine | Variable |
| 6 | 0.935 | 0.915 | DR, DM course, Hb, PP, sCr, sudden onset of heavy proteinuria |
| 7 | 0.943 | 0.930 | DR, DM course, Hb, PP, sCr, ALB, sudden onset of heavy proteinuria |
| 8 | 0.948 | 0.936 | DR, DM course, Hb, PP, sCr, ALB, TC, sudden onset of heavy proteinuria |
| 9 | 0.949 | 0.943 | DR, DM course, Hb, PP, sCr, ALB, TC, sudden onset of heavy proteinuria, hematuria |
| 10 | 0.953 | 0.947 | DR, DM course, Hb, PP, sCr, ALB, TC, sudden onset of heavy proteinuria, hematuria, family history of DM |
| 11 | 0.956 | 0.948 | DR, DM course, Hb, PP, sCr, ALB, TC, sudden onset of heavy proteinuria, age, hematuria, family history of DM |
| 12 | 0.955 | 0.952 | DR, DM course, Hb, PP, sCr, ALB, TC, 24hpro, sudden onset of heavy proteinuria, age, hematuria, family history of DM |

24hpro, 24-hr proteinuria; ALB, albumin; BUA, blood uric acid; DR, diabetic retinopathy; eGFR, estimated glomerular filtration rate; FBG, fasting blood glucose; MAP, mean arterial pressure; PP, pulse pressure; SBP, systolic blood pressure; TC, total cholesterol

| **Supplementary Table 7. Clinical parameters of new patients for external validation** | | | | |
| --- | --- | --- | --- | --- |
| Variables | DN(n=148) | NDRD(n=109) | Mixed(n=72) | *P*-value |
| Sex (male = 1) | 114(77.0%) | 73(67.0%) | 48(66.7%) | 0.127 |
| Age (year) | 51.83±9.66 | 51.16±9.64 | 53.63±8.93 | 0.288 |
| SBP (mmHg) | 176.41±25.00 | 158.10±25.60 | 166.13±32.43 | <0.001 |
| DBP (mmHg) | 102.41±19.63 | 99.08±17.35 | 95.51±18.42 | 0.056 |
| Pulse pressure (mmHg) | 74.00±18.81 | 60.22±21.52 | 70.61±23.17 | <0.001 |
| HBP (%) | 143(96.6%) | 90(82.6%) | 57(79.2%) | <0.001 |
| NS (%) | 44(29.7%) | 36(33.0%) | 35(48.6%) | 0.020 |
| DR (%) | 128(86.5%) | 24(22.0%) | 40(55.6%) | <0.001 |
| CCVD (%) | 71(48.0%) | 42(38.5%) | 24(33.3%) | 0.085 |
| Smoke (%) | 73(49.3%) | 47(43.1%) | 31(43.1%) | 0.529 |
| Drink alcoholic beverages (%) | 48(32.4%) | 40(36.7%) | 23(31.9%) | 0.725 |
| Course of diabetes (m) | 156.00(120.00,216.00) | 30.00(12.00,84.00) | 108(60.00,156.00) | <0.001 |
| Course of hypertension (m) | 19.00(2.00,96.00) | 36.00(0.00,120.00) | 24.00(0.00,120.00) | <0.840 |
| HbA1c(mmol/mol) | 56.54±17.28 | 51.20±14.52 | 51.67±16.13 | 0.016 |
| HbA1c (%) | 7.32±1.58 | 6.84±1.33 | 6.88±1.48 | 0.016 |
| FBG (mmol/L) | 6.48±2.75 | 6.44±2.16 | 6.07±2.32 | 0.261 |
| Hb (g/L) | 112.01±21.99 | 130.68±25.17 | 122.22±27.50 | <0.001 |
| BMI (kg/m^2^) | 26.42±3.75 | 26.82±4.88 | 26.43±5.02 | 0.585 |
| ALB (g/L) | 32.55±6.75 | 34.23±8.82 | 29.69±8.92 | 0.002 |
| 24-hr proteinuria (g/24h) | 3.86(2.16,5.82) | 2.49(0.88,5.00) | 3.67(2.32,5.83) | <0.001 |
| eGFR (mL/min1#73/m^2^) | 39.50(27.27,66.45) | 73.29(46.58,96.36) | 56.14(33.82,87.64) | <0.001 |
| BUA (µmol/L) | 376.31±90.74 | 382.82±105.91 | 371.07±94.64 | 0.785 |
| TC (mmol/L) | 4.80±1.60 | 5.24±2.10 | 6.02±2.61 | 0.002 |
| Triglyceride (mmol/L) | 2.19±1.71 | 2.96±2.70 | 2.75±1.59 | <0.001 |
| sCr (µmol/L) | 152.15(106.80,216.33) | 93.30(74.70,135.00) | 113.05(79.75,187.555) | <0.001 |
| Family history of DM (%) | 61(41.2%) | 27(24.8%) | 37(51.4%) | 0.001 |
| Family history of HBP (%) | 37(25.0%) | 34(31.2%) | 21(29.2%) | 0.532 |
| Sudden onset of heavy proteinuria (%) | 52(35.1%) | 56(51.4%) | 46(63.9%) | <0.001 |
| Hematuria (%) | 59(39.9%) | 62(56.9%) | 37(51.4%) | 0.021 |
| Systemic disease (%) | 19(12.8%) | 6(5.5%) | 5(6.9%) | 0.100 |

ALB, albumin; BMI, body mass index; BUA, blood uric acid; CCVDs, cardiovascular and cerebrovascular diseases; DBP, diastolic blood pressure; DR, diabetic retinopathy;

eGFR, estimated glomerular filtration rate; FBG, fasting blood glucose; HbA1c, hemoglobin A1C; MAP, mean arterial pressure; NS, nephrotic syndrome; SBP, systolic blood pressure

| **Supplementary Table 8. Comparison of performance among four models** | | | | | | | | |  |
| --- | --- | --- | --- | --- | --- | --- | --- | --- | --- |
|  | Isolated DN vs. isolated NDRD | | | | Isolated DN vs. non-DN | | | |  |
|  | SVM | RF | Model-2008 | Model-2014 | SVM | RF | Model-2008 | Model-2014 | |
| SVM |  | 0.044 | 4.899e-12 | 6.491e-05 |  | 0.082 | < 2.200e-16 | 6.030e-09 | |
| RF |  |  | <2.200e-16 | 1.774e-09 |  |  | < 2.200e-16 | 5.001e-14 | |
| Model-2008 |  |  |  | 0.004 |  |  |  | 1.330 e-4 | |

RF, random forest; SVM, support vector machine; DN, diabetic nephropathy; NDRD, non-diabetic renal disease;

| **Supplementary Table 9. Chi-squared Test for each model between two conditions** | | |
| --- | --- | --- |
| Models | Conditions | P-values |
| RF | Isolated DN vs. isolated NDRD | 0.914 |
|  | Isolated DN vs. non-DN |  |
| SVM | Isolated DN vs. isolated NDRD | 0.511 |
|  | Isolated DN vs. non-DN |  |
| Model-2008 | Isolated DN vs. isolated NDRD | 0.388 |
|  | Isolated DN vs. non-DN |  |
| Model-2014 | Isolated DN vs. isolated NDRD | 0.695 |
|  | Isolated DN vs. non-DN |  |

RF, random forest; SVM, support vector machine; DN, diabetic nephropathy; NDRD, nondiabetic renal disease; non-DN, NDRD patients and DN combined with NDRD patients.

| Supplementary table 10. The published models for differential diagnosis of DN and NDRD in recent years. | | | | | |
| --- | --- | --- | --- | --- | --- |
| Author | Jianhui Zhou[1] | Liu Mo-yan[2] | Zhenhua Y ang[3] | Shimin Jiang[4] | Florencio García-Martín[5] |
| Pubilication time | 2008 | 2014 | 2019 | 2019 | 2020 |
| country | China | China | China | China | spain |
| **statistical method** | logistic regression analysis. | Logistic regression, net reclassification improvement (NRI), and integrated discrimination  improvement (IDI) analysis | Univariate and  multivariate logistic regression analyses | Multivariable logistic regression analyses | Binary logistic regression analysis |
| samples | 110 | 200 | 213 | 302 | 207 |
| Sample collection time | 1993-2003 | 2004-2012 | 2011-2017 | 2014-2019 | 2000- 2018 |
| DN proportion | 54.5% | 46.5% | 34.7% | 47.4% | 61％ |
| NDRD protortion | 45.5% | 53.5% | 61.0% | 52.6% | 39％ |
| Kidney biopsy | yes | yes | yes | yes | yes |
| External validation | yes | yes | yes | yes | no |
| Multi-center | no | no | no | no | no |
| indicators | Dm:diabetes duration  Bp: systolic blood pressure  Dr:diabetic retinopathy  Hu:haematuria  Gh：HbA1c | Dm::diabetes duration  Bp： systolic blood pressure  Dr:diabetic retinopathy  Gh： HbA1c  Hu： haematuria  Hb:hemoglobin | DR  Proteinuria  Anemia  eGFR  DM  Bp | gender,  diabetes duration, diabetic retinopathy,  hematuria,  glycated hemoglobin A1c,  anemia,  blood pressure,  urinary protein excretion,  estimated glomerular filtration rate | presence of DR, insulin therapy,  duration of diabetes in therapy,CLLI, nephrotic proteinuria,  overweight  and microhematuria (a, therapy,ratethy,esd inte |
| models | P_DN_= exp(−13.5922 + 0.0371Dm+ 0.0395Bp + 0.3224Gh − 4.4552Hu + 2.9613Dr)/[1+exp(−13.5922+0.0371Dm+0.0395Bp+0.3224Gh− 4.4552Hu + 2.9613Dr)] | P_DN_= exp (0.846 + 0.022 Dm + 0.033Bp + 2.050 Gh–2.664Hu–0.078 Hb + 2.942Dr)/[1 + exp (0.846 + 0.022 Dm + 0.033 Bp + 2.050Gh–2.664 Hu–0.078 Hb + 2.942 Dr)]. | P_NDRD_=1/[1+exp(−17.382–3.339×DR  −1.274×Proteinuria−2.217×Anemia-1.853×eGFR−0.993×DM+20.892Bp)]. | no | no |
| Whether Mixed group included when modeling | exclude | exclude | include | exclude | exclude |
| ROC AUC | 0.968 | 0.971. | 0.93 | 0.934 | 0.92 |
| External validation | 90.5%(Total consistency) | 90.9%(Total consistency) | 90.60%（Total consistency） | 0.875（The C-index value） | 无外部验证 |
| NDRD constitution | IgA nephropathy(34%)，Membranous nephropathy(22%)， mesangial proliferative glomerulonephritis (14%，exclude IgA nephropathy) | IgA nephropathy(32.7%)；Membranous nephropathy (18.7%)； Obesity-related glomerulopathy (7.5%)，Focal segmental glomerular sclerosis(7.5%)； mesangial proliferative glomerulonephritis (5.6%)，others(22.4%)。 | Idiopathic membranous nephropathy(46%) focal segmental glomerulosclerosis(15%)、IgA nephropathy(14%)、others | IgA nephropathy(20.1%)， Hepatitis B virus-associated glomerulonephritis (27%)，Membranous nephropathy(18.2%)， Obesity-related glomerulopathy (8.8%)， Acute tubular interstitial disease of kidney (6.3%)， Focal segmental glomerular sclerosis (5.7%)， minimal change nephrosis (3.8%) | primary glomerular diseases (52％), nephro-angiosclerosis (16％), Immunoallergic interstitial nephritis (15％), vasculitis (8.5％),other |

| **Supplementary Table 11. Performance for SVM and RF established with population (2015-2017) in the external validation** | | | | | | |
| --- | --- | --- | --- | --- | --- | --- |
|  | Models | Sensitivity | Specificity | PPV | NPV | ROC AUC |
| Isolated-DN vs. Isolated NDRD | RF | 0.862 | 0.838 | 0.885 | 0.807 | 0.909 |
|  | SVM | 0.887 | 0.809 | 0.851 | 0.853 | 0.896 |
| Isolated-DN vs. non-DN | RF | 0.758 | 0.818 | 0.784 | 0.796 | 0.841 |
|  | SVM | 0.707 | 0.865 | 0.865 | 0.707 | 0.836 |

RF, random forest; SVM, support vector machine; DN, diabetic nephropathy; NDRD, nondiabetic renal disease; non-DN, NDRD patients and DN combined with NDRD patients.

## Figures captions

**Supplementary Figure 1.** ROC AUC of support vector machine (SVM) and random forest (RF) methods with different number of variables.

**Supplementary Figure 2. The area under the receiver operating characteristic curve (AUC ROC) for random forest (RF), support vector machine (SVM) and logistic regression methods of external validation.** A: The ROC for four models under ideal condition (isolated DN vs. isolated DN patients). The AUC ROC for RF was 0.920. The cut-off value point was A (0.899, 0.872), with a sensitivity of 0.905, specificity of 0.864, positive predictive value (PPV) of 0.899, and negative predictive value (NPV) of 0.872. The AUC ROC for the logistic regression method (model-2008) was 0. 886. The cut-off value point was B (0.835,0.851), with a sensitivity of 0.893, specificity of 0.706, PPV of 0.730, and NPV of 0.881. The AUC ROC for the logistic regression method (model-2014) was 0.917. The cut-off value point was C (0.862,0.858), with a sensitivity of 0.858, specificity of 0.853, PPV of 0.899, and NPV of 0.798. The AUC ROC for SVM was 0.911. The cut-off value point was D (0.926, 0.807), with a sensitivity of. 0.867, specificity of 0.889, PPV of 0.926, and NPV of 0.807. B: The ROC for four models under actual condition (isolated DN vs. non-DN patients). The AUC ROC for RF was 0.855. The cut-off value point was E (0.899, 0.735), with a sensitivity of.73.48%, specificity of 89.86%, PPV of 0.899, and NPV of 0.735. The AUC ROC for the logistic regression method (model-2008) was 0.821. The cut-off value point was F (0.663,0.885), with a sensitivity of 0.732, specificity of 0.765, PPV of 0.703, and NPV of 0.790. G: The AUC ROC for the logistic regression method (model-2014) was 0.841. The cut-off value point was G (0.707,0.858), with a sensitivity of 0.688, specificity of 0.892, PPV of 0.669, and NPV of 0.841. The AUC ROC for the SVM was 0. 846. The cut-off value point was H (0.892,0.713), with a sensitivity of 0.717, specificity of 0.890, PPV of 0.892, and NPV of 0.713.

## Supplementary Reference

1. Zhou, J., et al., *A differential diagnostic model of diabetic nephropathy and non-diabetic renal diseases.* Nephrol Dial Transplant, 2008. **23**(6): p. 1940-5.

2. Liu, M.Y., et al., *Validation of a differential diagnostic model of diabetic nephropathy and non-diabetic renal diseases and the establishment of a new diagnostic model.* J Diabetes, 2014. **6**(6): p. 519-26.

3. Yang, Z., et al., *A Differential Diagnosis Model For Diabetic Nephropathy And Non-Diabetic Renal Disease In Patients With Type 2 Diabetes Complicated With Chronic Kidney Disease.* Diabetes Metab Syndr Obes, 2019. **12**: p. 1963-1972.

4. Jiang, S., et al., *Novel Model Predicts Diabetic Nephropathy in Type 2 Diabetes.* Am J Nephrol, 2020. **51**(2): p. 130-138.

5. Garcia-Martin, F., et al., *When to perform renal biopsy in patients with type2 diabetes mellitus? Predictive model of non-diabetic renal disease.* Nefrologia (Engl Ed), 2020. **40**(2): p. 180-189.
